# Finite Element Investigation of the Influence of Strut Diameter on the Mechanical Performance of Balloon-Expandable Biodegradable PLA/PDO Coronary Stents

**DOI:** 10.3390/polym18172177

**Published:** 2026-09-07

**Authors:** Elhadj Besseghier, Fatima Zohra Kettaf, Ahmed Ouadah Bouakkaz, Abdelkader Djebli, Ali Benhamena, Dursun Murat Sekban, Ecren Uzun Yaylacı, Merve Terzi, Murat Yaylacı

**Affiliations:** 1 Mechanical Engineering Department, Faculty of Technology, Hassiba Benbouali University of Chlef, Hay Salem, National Road N19-02000, Chlef 02000, Algeria; e.besseghier@univ-chlef.dz (E.B.); a.bouakkaz@univ-chlef.dz (A.O.B.); 2 Mechanics of Materials, Energy and Environment Laboratory (L2M2E), University of Mascara, Mascara 29000, Algeria; djebliabdelkader@univ-mascara.dz (A.D.); ali.benhamena@univ-mascara.dz (A.B.); 3 Faculty of Mechanical Engineering, University of Science and Technology of Oran Mohamed-Boudiaf USTO-MB, Oran 31000, Algeria; fatimazohra.kettaf@univ-usto.dz; 4 Laboratory of Materials and Reactive Systems, University of Djillali Liabes, Sidi Bel Abbes 22000, Algeria; 5 Department of Marine Engineering Operations, Karadeniz Technical University, Trabzon 61080, Türkiye; msekban@ktu.edu.tr; 6 Trabzon Teknokent, WMS Engineering Services Industry Trade Limited Company, Trabzon 61080, Türkiye; 7 Faculty of Fisheries, Recep Tayyip Erdogan University, Rize 53100, Türkiye; ecren.uzunyaylaci@erdogan.edu.tr; 8 Department of Civil Engineering, Recep Tayyip Erdogan University, Rize 53100, Türkiye; merve_terzi21@erdogan.edu.tr; 9 Turgut Kıran Maritime Faculty, Recep Tayyip Erdogan University, Rize 53900, Türkiye; 10 Dijitalpark Teknokent, Murat Yaylacı-Luzeri R&D Engineering Company, Rize 53100, Türkiye

**Keywords:** PLA/PDO blend, biodegradable stent, finite element analysis, strut diameter, mechanical performance, deployment behavior, coronary stent

## Abstract

This study numerically investigates the influence of strut diameter on the deployment behavior of a balloon-expandable biodegradable stent with rhombic cell architecture using finite element analysis. The stent material was represented by a 60/40 poly(lactic acid)/polydioxanone (PLA/PDO) blend. Four stent configurations with strut diameters of 0.15, 0.25, 0.35, and 0.50 mm were analyzed under identical deployment conditions. The numerical evaluation considered von Mises stress together with five deployment indicators: diametral strain, elastic recoil, dog-boning, foreshortening, and longitudinal retraction. The results show that increasing the strut diameter reduces elastic recoil, foreshortening, and longitudinal retraction, thereby enhancing post deployment dimensional stability. However, thicker struts also increase the dog-boning effect, indicating less uniform radial expansion. Among the investigated designs, the stent with a 0.35 mm strut diameter showed a balanced response between deployment uniformity and post deployment mechanical stability under the adopted numerical assumptions. For this configuration, elastic recoil, foreshortening, longitudinal retraction, and dog-boning were approximately 6.2%, 10%, 37%, and 8.9%, respectively. These findings provide practical design guidance for biodegradable polymeric vascular stents.

## 1. Introduction

Cardiovascular diseases (CVDs), particularly coronary artery disease caused by atherosclerotic plaque formation, remain one of the leading causes of morbidity and mortality worldwide [1,2,3]. Percutaneous coronary intervention (PCI) combined with stent implantation has become the standard therapeutic approach for restoring vessel patency and improving myocardial perfusion in patients with coronary artery stenosis [4,5,6]. Over the past decades, coronary stents have evolved from bare-metal stents (BMSs) to drug-eluting stents (DESs), significantly reducing restenosis rates and improving long-term clinical outcomes [7,8]. Despite these advances, several post-deployment complications, including in-stent restenosis, late stent thrombosis, and mechanical failure such as stent fracture, continue to limit the long-term effectiveness of these devices [9,10,11]. The mechanical behavior of a stent during and after deployment plays a fundamental role in determining its clinical performance. An ideal stent should provide sufficient radial support to maintain vessel patency while minimizing injury to the arterial wall during expansion. Consequently, the mechanical performance of balloon-expandable stents is commonly evaluated using several key performance indicators, including diametral strain, elastic recoil, dog-boning, foreshortening, longitudinal retraction, and radial stiffness [12,13,14]. These parameters characterize the expansion capability, deployment uniformity, post-deployment dimensional stability, and structural integrity of the stent. Excessive elastic recoil reduces the final lumen diameter and compromises the immediate success of the intervention, whereas pronounced dog-boning may generate localized stress concentrations at the stent ends, increasing the risk of arterial injury. Likewise, excessive foreshortening and longitudinal retraction reduce deployment accuracy and may negatively affect lesion coverage [15,16]. Among the various design parameters influencing these mechanical characteristics, the strut diameter is one of the most critical geometric factors. Increasing the strut diameter generally enhances the radial stiffness and mechanical stability of the stent, thereby reducing elastic recoil and improving its ability to preserve the expanded diameter after balloon deflation. However, thicker struts may also increase deployment non-uniformity, reduce flexibility, and intensify the dog-boning effect. Conversely, thinner struts improve flexibility and conformability but may exhibit larger elastic recovery and lower radial support [17,18,19,20]. Therefore, the selection of an appropriate strut diameter requires a careful balance between these competing mechanical requirements. For biodegradable polymeric stents, this balance is particularly important because deployment behavior depends on both material response and scaffold geometry. Finite element analysis (FEA) has become an indispensable tool for investigating the biomechanical behavior of vascular stents because it provides detailed information on stress distribution, deformation patterns, and structural response throughout the deployment process [21,22,23,24,25,26,27]. Numerous numerical studies have employed FEA to evaluate the influence of cell geometry, material properties and deployment conditions on stent performance [28,29,30,31]. These investigations have demonstrated that geometric modifications significantly affect stress concentration, plastic deformation, and the overall mechanical response of the stent. Although considerable attention has been devoted to optimizing cell topology, connector design, and material selection, relatively few studies have systematically investigated the influence of strut diameter on multiple deployment performance indicators, particularly for biodegradable stents with rhombic-cell geometry. Several published works evaluate only one or two mechanical parameters, such as elastic recoil, radial strength, or dog-boning, without providing a comprehensive assessment of their coupled effects on deployment behavior and post-deployment stability [32,33]. Consequently, the relationship between strut diameter and the overall mechanical performance of rhombic-cell biodegradable stents remains insufficiently understood. Therefore, the objective of this study is to numerically investigate the influence of strut diameter on the deployment behavior of a balloon-expandable biodegradable PLA/PDO stent with a rhombic-cell architecture using finite element analysis. Four stent configurations with different strut diameters were analyzed under identical deployment conditions. The mechanical performance was evaluated through five key performance indicators: diametral strain, elastic recoil, dog-boning, foreshortening, and longitudinal retraction. Based on the combined analysis of these indicators, the study aims to identify the strut diameter that provides the most favorable compromise between deployment uniformity and post-deployment mechanical stability, thereby providing practical design guidelines for the development of next-generation biodegradable vascular stents.

## 2. Materials and Methods

### 2.1. Stent Geometry and Finite Element Model

**Figure 1:**
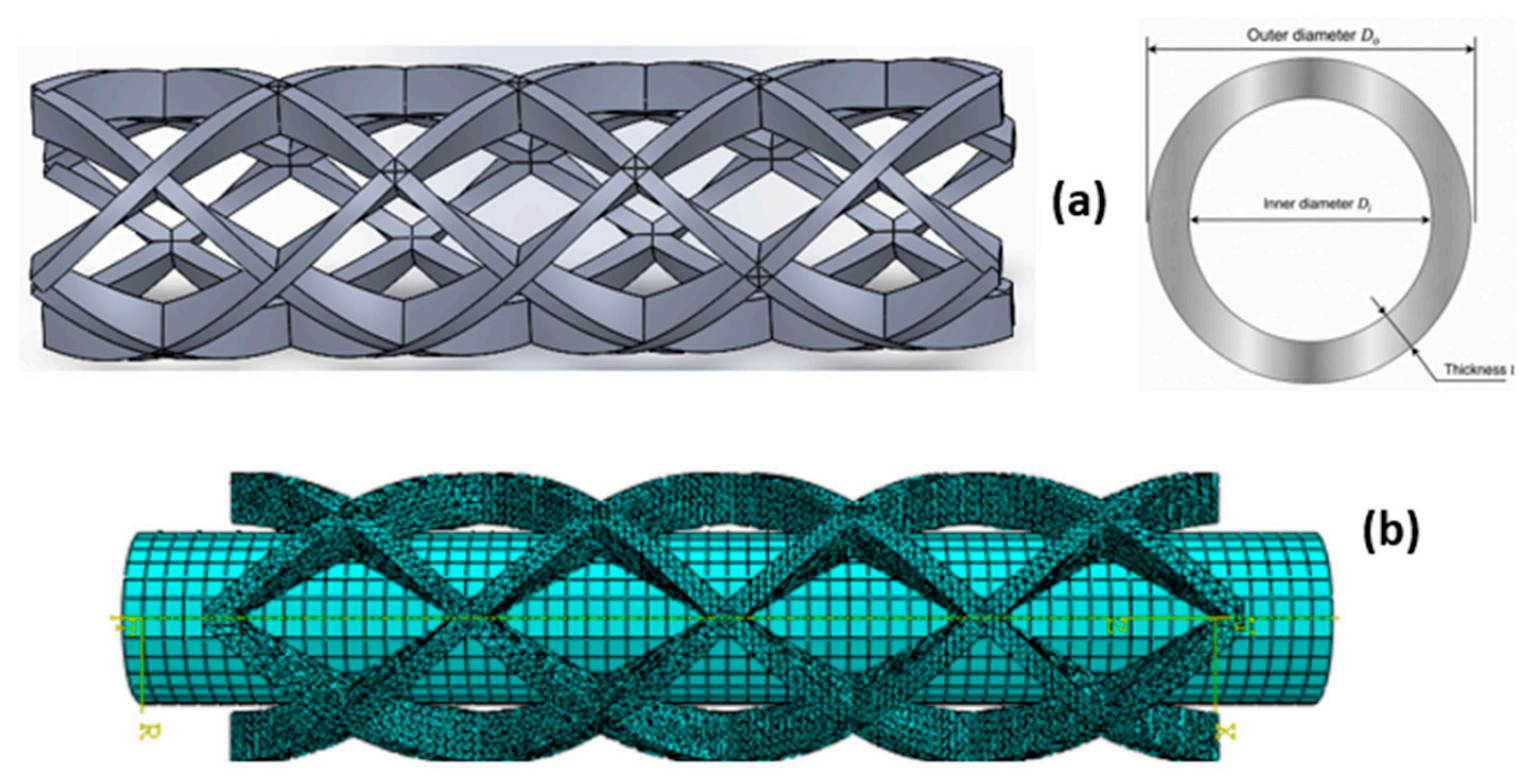
Geometry and finite element model of the balloon–stent system: (**a**) SolidWorks model of the stent with the strut cross-section; (**b**) three-dimensional finite element mesh of the balloon–stent assembly.

The baseline balloon-expandable stent geometry adopted in this study is shown in Figure 1a. The reference stent consists of a repeating diamond-cell architecture with rectangular struts, representing the original design selected for numerical validation. The stent has an overall length of Ls = 10 mm, an outer diameter of Do = 3 mm, and Np = 4 peaks. Each strut is characterized by a rectangular cross-section with a width of w = 0.201 mm and a thickness of t = 0.5 mm. The three-dimensional geometry was created using SolidWorks 2025 (Dassault Systèmes SolidWorks Corporation, Waltham, MA, USA) and subsequently imported into Abaqus/Explicit 2020 (Dassault Systèmes Simulia Corp., Providence, RI, USA) for finite element analysis. This reference configuration served as the baseline model for validation against the numerical results reported by Okereke [13].

Figure 1b presents the finite element discretization of the stent–balloon assembly. Due to its relatively small wall thickness, the balloon was modeled using SFM3D8 eight-node quadrilateral surface elements with a mesh size of 0.2 mm, whereas the stent was meshed using C3D10 ten-node tetrahedral solid elements with a mesh size of 0.1 mm. The adopted 0.1 mm quadratic stent mesh was additionally checked through the baseline validation, which reproduced the reference deformation histories and yielded a peak von Mises stress difference of 5.8%. The same C3D10 discretization protocol was retained for all four stent configurations to provide a consistent basis for the parametric comparison.

The stent material considered in the present numerical model was a PLA/PDO (60/40) blend. The material properties and post-yield response were adopted from the experimentally characterized PLA/PDO formulation reported by Okereke et al. [13]. In that study, medical-grade polydioxanone (PDO, RESOMER X206S) and polylactic acid (PLA, RESOMER L206S) were obtained from Evonik, Darmstadt, Germany. The corresponding material properties used in the present model are summarized in Table 1. The PLA/PDO stent was modeled as a homogeneous, isotropic elastoplastic material, whereas the balloon was modeled as homogeneous, isotropic, and linearly elastic. For the stent, the Young’s modulus and Poisson ratio in Table 1 define the initial elastic response, and the post-yield branch follows the experimentally characterized 60/40 PLA/PDO response used in the reference model. Material damage and failure were not included in the present analysis.

### 2.2. Loading and Boundary Conditions

To apply the loading conditions, a cylindrical coordinate system was defined, where the r-axis corresponds to the radial expansion direction, the θ-axis represents the circumferential direction around the stent, and the *z*-axis denotes the longitudinal axis of the stent. Balloon expansion was simulated by prescribing a time-dependent radial displacement on the balloon surface over a total simulation time of 1 s. The loading history consisted of three successive stages: (i) inflation from t = 0 s to t = 0.45 s, during which the radial displacement increased linearly until full balloon expansion was achieved; (ii) a holding stage between t = 0.45 s and t = 0.55 s, during which the balloon remained fully inflated to ensure complete stent deployment; and (iii) deflation from t = 0.55 s to t = 1 s, during which the prescribed radial displacement was gradually removed, allowing the balloon to collapse and the stent to undergo elastic recoil. Rigid-body motion was suppressed by constraining axial translation of the assembly and fixing the circumferential degree of freedom at a reference node, while radial deformation remained unrestricted. The balloon–stent interaction was defined using finite-sliding surface-to-surface contact with hard normal contact and a penalty tangential formulation with a friction coefficient of 0.2.

**Figure 2:**
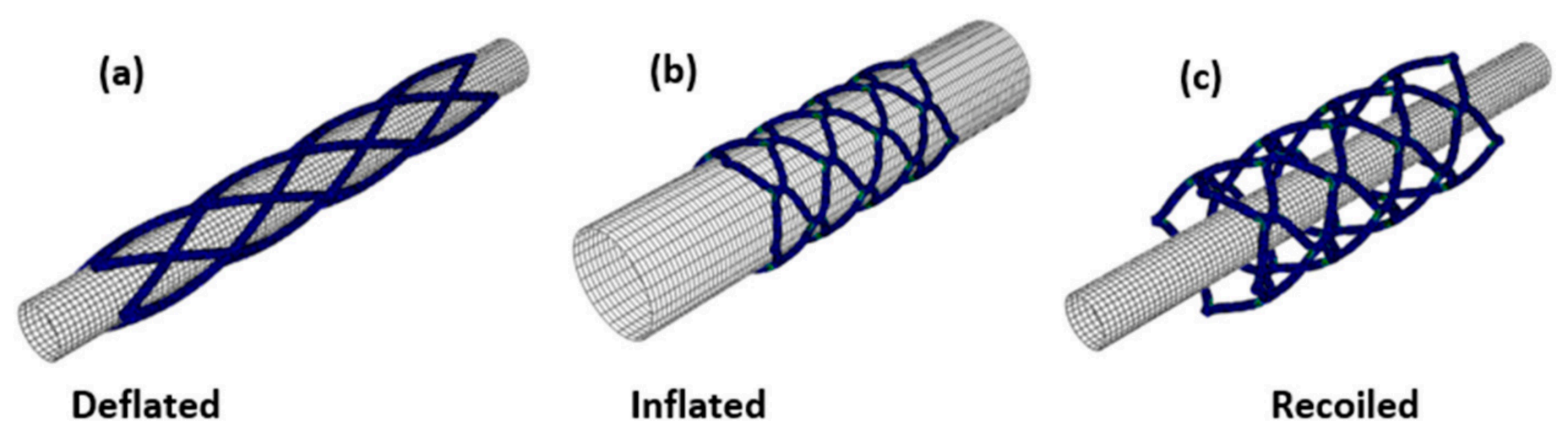
Deployment process of the stent: (**a**) deflated state, (**b**) inflated state, and (**c**) recoiled state after balloon deflation.

Figure 2 illustrates the three principal stages of the balloon-expandable stent deployment process considered in the finite element simulation. At t = 0 s, the balloon is in the deflated state and the stent remains in its initial crimped configuration (Figure 2a). During the holding stage (t = 0.45–0.55 s), the balloon reaches its maximum expansion, resulting in complete stent deployment (Figure 2b). Following balloon deflation, the stent experiences elastic recoil and reaches its final deployed configuration at t = 1 s (Figure 2c).

The numerical model was established under the following assumptions: the effect of blood pressure was neglected; the tri-folded configuration of the balloon in the deflated state was not modeled; and the balloon was assumed to maintain a perfect cylindrical shape throughout the simulation. The arterial wall was also excluded from the model. These assumptions isolate the geometric influence of strut diameter while excluding vessel confinement and material rate effects from the simulated deployment response. Accordingly, the absence of arterial-wall confinement and blood pressure may influence the magnitude of radial recoil, while the idealized cylindrical balloon may affect the end-expansion behavior and the resulting dog-boning response. In addition, the adopted elastoplastic material representation does not account for viscoelastic rate-dependent recovery during the 1 s loading cycle. Therefore, the model is intended primarily for controlled comparison of the investigated strut geometries rather than direct prediction of in vivo deployment behavior.

### 2.3. Structural Performance Evaluation Parameters

To evaluate the structural performance of the stent after deployment, four output parameters were considered: elastic recoil, longitudinal retraction, foreshortening, and dog-boning [13]. Elastic recoil was defined as the percentage reduction in stent diameter from the fully inflated state to the recoiled state after balloon deflation, reflecting the elastic recovery of the stent. Longitudinal retraction was calculated as the percentage change in stent length between the deflated and fully inflated states, providing a measure of the axial shortening that occurs during expansion. Foreshortening was quantified as the percentage difference in stent length between the recoiled and fully inflated states, representing the axial deformation caused by elastic recovery after balloon deflation. Finally, the dog-boning effect was evaluated by comparing the stent diameter at the ends with that at the center after expansion. This parameter quantifies the degree of non-uniform radial expansion and is expressed as the percentage difference between the end diameter and the central diameter of the stent. These four parameters provide a comprehensive assessment of the mechanical performance and deployment behavior of the stent.

## 3. Numerical Model Verification

**Figure 3:**
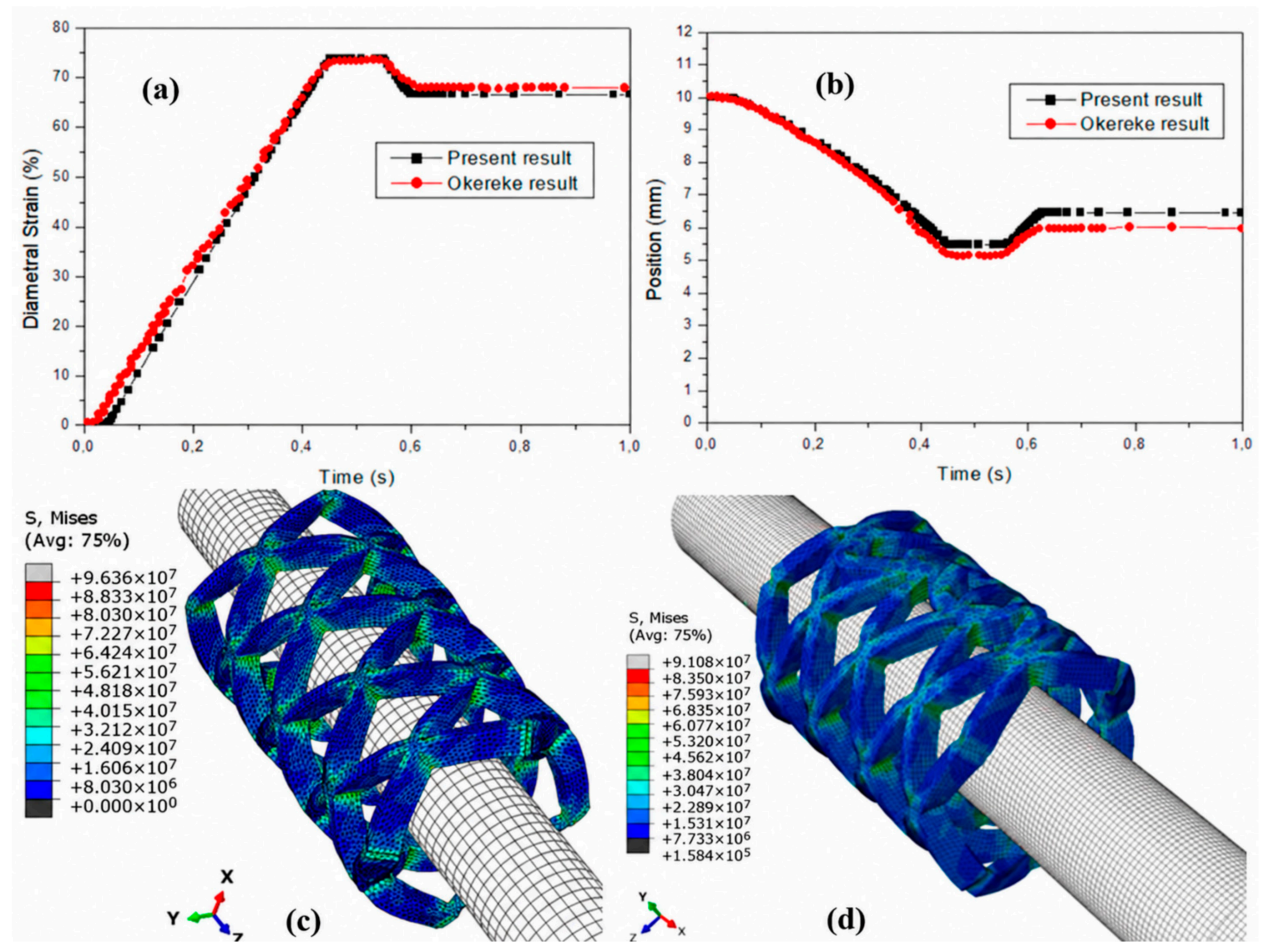
Comparison of the present finite element results with those of Okereke et al.: (**a**) diametral strain and (**b**) axial position versus time; (**c**) von Mises stress distribution predicted by the present model after balloon expansion and recoil; and (**d**) von Mises stress distribution reported by Okereke.

To verify the consistency of the developed finite element (FE) model, the predicted diametral strain, axial position, and von Mises stress distribution were compared with the published numerical results reported by Okereke et al. [13]. As shown in Figure 3, the present model exhibits close agreement with the reference model throughout both the expansion and recoil stages.

The diametral strain increases almost linearly during balloon expansion, reaching a maximum value of approximately 73% at about 0.45 s, followed by a slight decrease and stabilization due to elastic recoil. Likewise, the axial position decreases progressively during expansion to approximately 5 mm, before partially recovering after balloon deflation, reproducing the same trend reported by Okereke. The minor differences observed after approximately 0.6 s are small and may be attributed to variations in mesh density, contact formulation, numerical stabilization techniques, or material implementation. In addition to this quantitative comparison, the similarity in the von Mises stress distributions provides further evidence of numerical consistency between the two models. Both models exhibit similar stress localization patterns, with the highest stresses concentrated at the strut crowns and connecting bridge regions, while relatively lower stresses are observed along the straight sections of the struts. Furthermore, the maximum von Mises stress predicted by the present model is 96.36 MPa, compared with 91.08 MPa reported by Okereke, corresponding to a difference of only 5.8%. This discrepancy indicates close correspondence between the two numerical models and may be associated with minor differences in discretization, contact algorithms, and numerical solution procedures. Overall, the agreement in the deformation histories and stress localization supports the numerical consistency of the implemented FE formulation and its use for the subsequent controlled parametric comparison. This comparison is intended as a numerical verification against a published reference model and should not be interpreted as experimental validation of the physical accuracy of the model. The close recoil-stage agreement is consistent with adopting the same elastoplastic constitutive class and 60/40 PLA/PDO response as the reference model.

**Figure 4:**
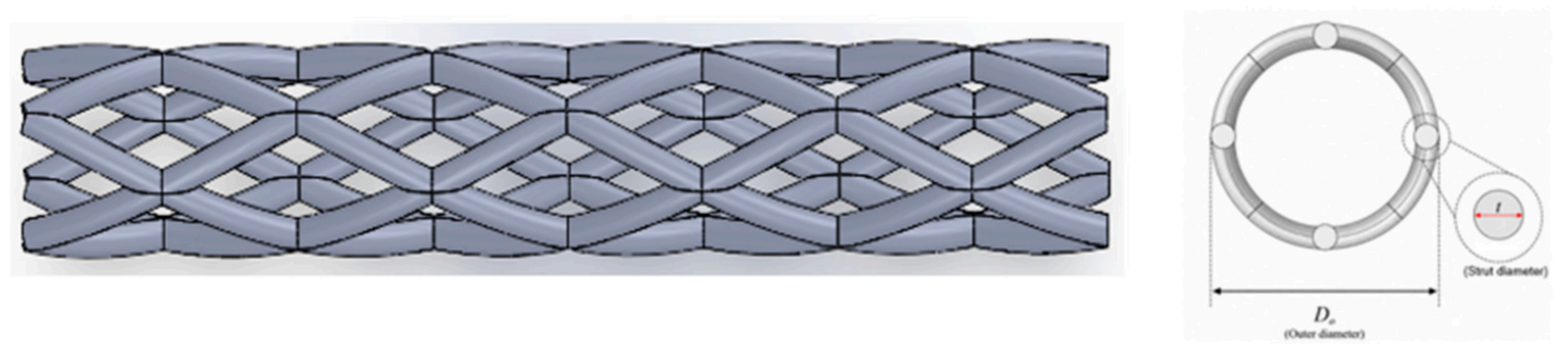
Geometric model of the stent used for the parametric study, illustrating the outer stent diameter (Do) and circular strut diameter (t).

Following this numerical verification, the alternative stent design shown in Figure 4 was introduced for the parametric study. The 10 mm reference geometry used for numerical verification reproduces the configuration reported by Okereke et al. and uses rectangular struts. The subsequent parametric study uses a separate 20 mm rhombic-cell geometry with circular struts; its length and topology were held constant while only the circular strut diameter was varied.

The geometric characteristics of the stent and balloon models are listed in Table 2 and Table 3. The objective of this study was to investigate the influence of the strut profile diameter (thickness) on the mechanical performance of the balloon-expandable stent during deployment. The analysis focused on the evolution of the expansion behavior, von Mises stress, dog-boning, elastic recoil, longitudinal retraction, and foreshortening as the strut profile diameter was varied.

## 4. Results and Discussion

### 4.1. Von Mises Stress Distribution

**Figure 5:**
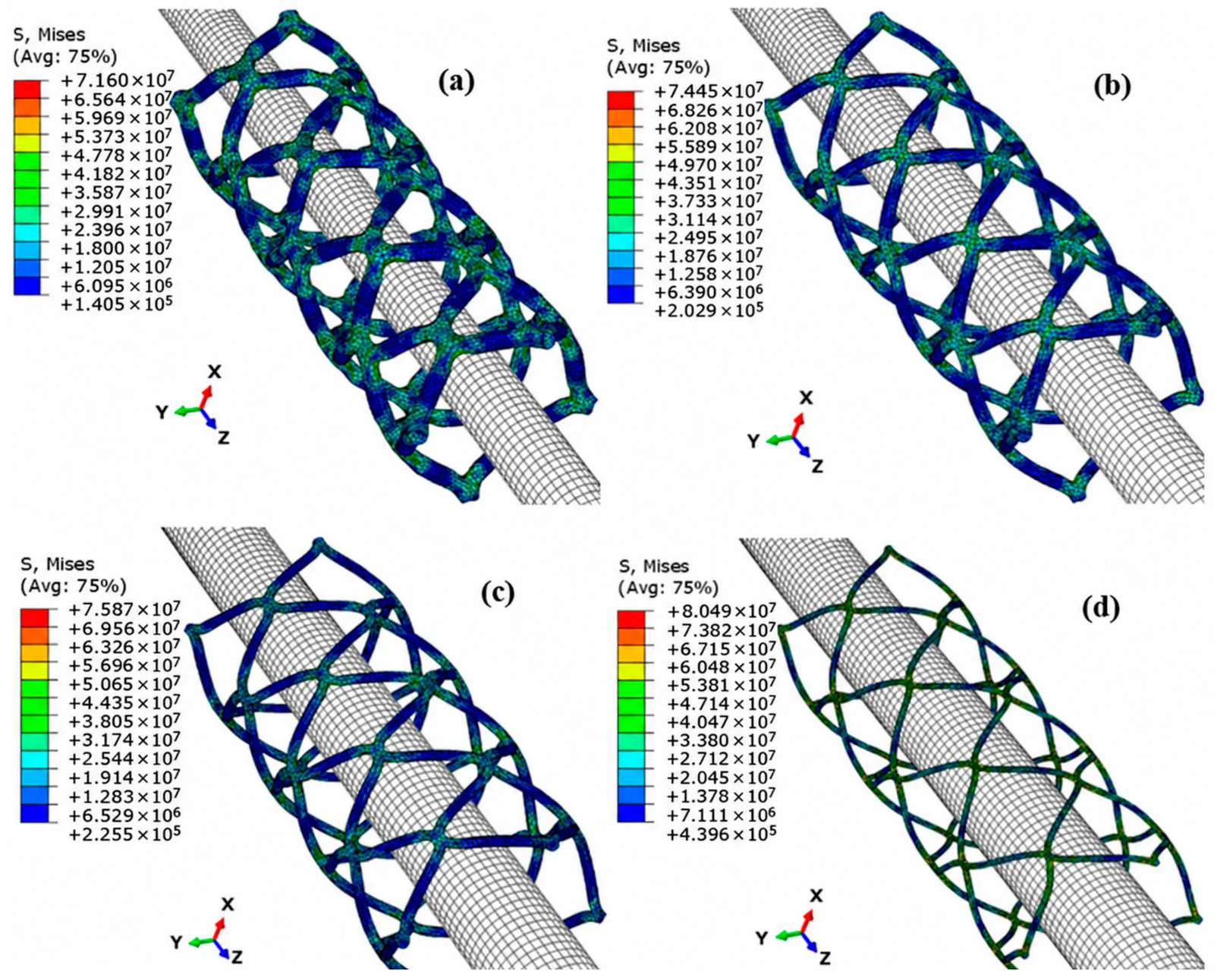
Effect of strut diameter on the von Mises stress distribution in the stent after balloon expansion and recoil: (**a**) t = 0.50 mm, (**b**) t = 0.35 mm, (**c**) t = 0.25 mm, and (**d**) t = 0.15 mm.

Figure 5 presents the von Mises stress distributions during balloon expansion for stents with strut diameters of 0.50, 0.35, 0.25, and 0.15 mm. In all configurations, the highest stresses are consistently concentrated at the crown regions and connecting bridges, where the largest bending deformation occurs during expansion. A clear reduction in the maximum von Mises stress is observed as the strut diameter increases. Specifically, the peak stress decreases from approximately 80.49 MPa for the 0.15 mm strut to 71.90 MPa for the 0.50 mm strut, corresponding to an overall reduction of approximately 10.7%. This behavior is attributed to the increased cross-sectional area and flexural rigidity of thicker struts, which distribute the applied load more efficiently and reduce the stress levels within the scaffold. Moreover, the stress contours become more homogeneous with increasing strut diameter, indicating a more uniform load transfer within the scaffold. Because material damage and failure were not included in the constitutive model, the reported stress values were used for relative comparison among the configurations and should not be interpreted as failure predictions.

### 4.2. Deployment and Structural Performance

**Figure 6:**
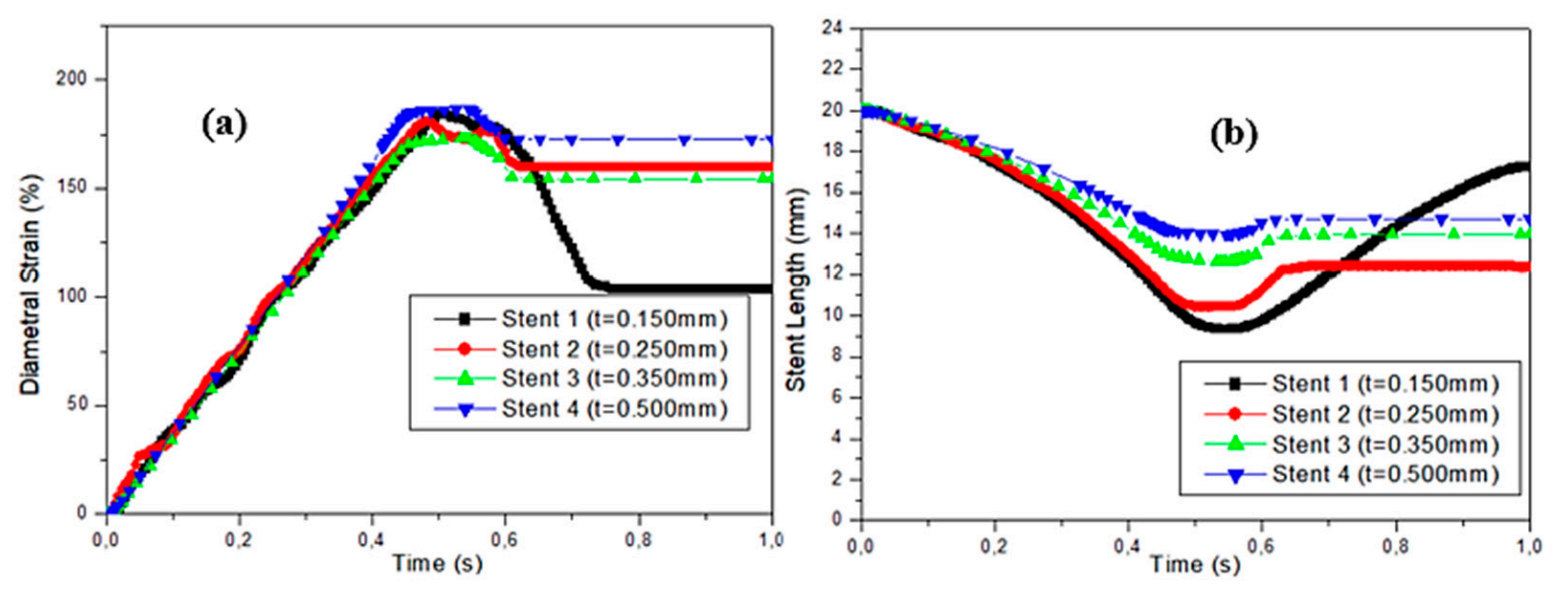
Effect of strut diameter on the deployment behavior of the stent: (**a**) diametral strain evolution and (**b**) stent length variation as a function of time during balloon expansion and recoil.

Figure 6a shows the evolution of diametral strain as a function of time for the four stent models with different strut diameters. During the inflation stage (0–0.45 s), the diametral strain increased almost linearly for all stent models, indicating a uniform response of the stents to the applied radial balloon displacement. As the inflation process continued, the diametral strain reached its maximum value at approximately 0.5 s. The stent with a 0.50 mm strut diameter exhibited the highest peak diametral strain, followed by the 0.15 mm stent, whereas the 0.25 mm and 0.35 mm stents showed slightly lower and comparable peak values.

Following the onset of balloon deflation (0.55 s), the diametral strain decreased in all stents due to elastic recoil. However, the magnitude of this reduction depended on the strut diameter. The 0.15 mm stent exhibited the greatest decrease, with the diametral strain stabilizing at approximately 105%, indicating the highest radial recoil. In contrast, the 0.50 mm stent retained the highest final diametral strain (approximately 170%) after balloon withdrawal, demonstrating the lowest radial recoil and the greatest ability to maintain the expanded diameter. The 0.25 mm and 0.35 mm stents exhibited intermediate behavior, with final diametral strain values of approximately 155% and 160%, respectively. These results indicate that increasing the strut diameter improves the post-deployment stability of the stent and reduces radial recoil, owing to the increase in structural rigidity. For the rhombic-cell stent design, a larger strut diameter limits strut rotation during unloading, thereby helping to preserve the final expanded diameter and minimizing the loss of expansion caused by elastic recoil. This behavior is consistent with the fundamental mechanical principles governing vascular stents, whereby thicker struts provide greater resistance to radial recoil due to their higher structural rigidity. However, this improvement is generally accompanied by reduced flexibility and lower trackability through tortuous vascular pathways. Figure 6b presents the evolution of the stent axial length during the deployment process. During balloon inflation (0–0.55 s), the stent length decreased progressively for all models, indicating longitudinal shortening associated with radial expansion. The maximum shortening occurred at approximately 0.55 s, after which the stent length increased during balloon deflation due to elastic recovery. However, the extent of recovery depended on the strut diameter. The 0.15 mm stent exhibited the greatest recovery, reaching a final length of approximately 17 mm, whereas the 0.50 mm stent showed the smallest recovery and stabilized at approximately 14.5 mm. The 0.25 mm and 0.35 mm stents displayed intermediate behavior. These results indicate that increasing the strut diameter enhances the axial stiffness of the rhombic-cell stent, thereby limiting longitudinal recovery after deployment.

**Figure 7:**
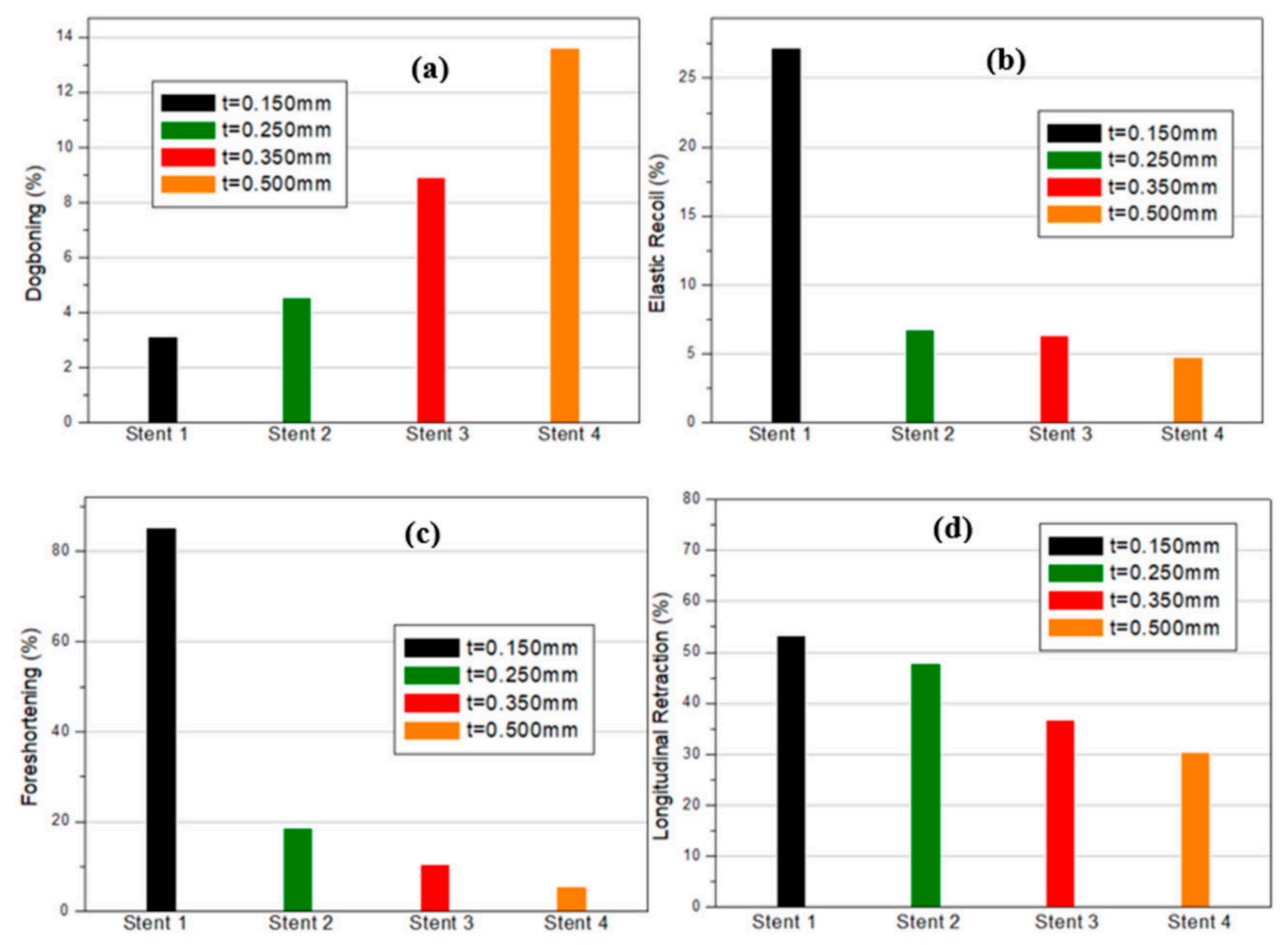
Effect of strut diameter on the structural performance parameters of the stent after deployment: (**a**) dog-boning, (**b**) elastic recoil, (**c**) foreshortening, and (**d**) longitudinal retraction.

The four structural performance parameters obtained after deployment are compared in Figure 7. Figure 7a presents the dog-boning ratio of the four stent models with different strut diameters. The results reveal a clear increasing trend in dog-boning with increasing strut diameter. The dog-boning ratio increased from approximately 3.1% for the 0.150 mm stent to 4.5%, 8.9%, and 13.6% for the 0.250 mm, 0.350 mm, and 0.500 mm stents, respectively. This indicates that stents with thicker struts experience greater non-uniform radial expansion, with the end regions expanding more rapidly than the central section during balloon inflation. This behavior can be attributed to the increased structural stiffness associated with larger strut diameters. Thicker struts require higher deformation energy and exhibit greater resistance to uniform expansion, leading to preferential expansion at the less constrained ends before the central region fully expands. Consequently, increasing the strut diameter promotes a more pronounced dog-boning effect in rhombic-cell stents. Excessive dog-boning is generally undesirable because it may generate localized stress concentrations at the stent ends and increase the risk of arterial wall injury during deployment. Therefore, although increasing the strut diameter reduces radial recoil, it also intensifies the dog-boning phenomenon, highlighting the trade-off that must be considered during stent design optimization.

Figure 7b illustrates the elastic recoil of the four stent models with different strut diameters after balloon deflation. The results demonstrate a significant reduction in elastic recoil with increasing strut diameter. The 0.150 mm stent exhibited the highest elastic recoil of approximately 27%, whereas the recoil decreased markedly to approximately 6.7%, 6.2%, and 4.8% for the 0.250 mm, 0.350 mm, and 0.500 mm stents, respectively. The substantial reduction in elastic recoil can be attributed to the increase in the structural rigidity of the stent as the strut diameter increases. Thicker struts possess greater bending resistance and structural rigidity, enabling the expanded configuration to be maintained more effectively after balloon deflation. Consequently, less elastic recovery occurs, resulting in improved dimensional stability and a larger final lumen diameter. For rhombic-cell stents, increasing the strut diameter also restricts strut rotation and deformation at the cell junctions, thereby reducing the tendency of the stent to recover elastically. Although this behavior is beneficial for minimizing recoil and maintaining vessel patency, excessively large strut diameters may adversely affect other performance indicators, such as flexibility and dog-boning. Therefore, the results highlight the design trade-off between minimizing elastic recoil and preserving favorable deployment characteristics.

Figure 7c presents the foreshortening of the four rhombic-cell stent models with different strut diameters. A pronounced reduction in foreshortening is observed as the strut diameter increases. The 0.150 mm stent exhibited the highest foreshortening of approximately 85%, whereas the values decreased substantially to about 18%, 10%, and 5% for the 0.250 mm, 0.350 mm, and 0.500 mm stents, respectively.

This trend indicates that increasing the strut diameter significantly improves the longitudinal dimensional stability of the stent during deployment. Thicker struts possess higher axial and bending stiffness, which limits the rotation and bending of the rhombic cells during radial expansion. Consequently, the longitudinal shortening associated with cell deformation is substantially reduced. In rhombic-cell stents, radial expansion is accompanied by a geometric reorientation of the struts, resulting in longitudinal shortening. Increasing the strut diameter restricts this geometric deformation and allows the stent to better preserve its original axial length.

Figure 7d presents the longitudinal retraction of the four rhombic-cell stent models with different strut diameters. The results show a progressive decrease in longitudinal retraction as the strut diameter increases. The 0.150 mm stent exhibited the highest longitudinal retraction of approximately 53%, followed by the 0.250 mm (48%), 0.350 mm (37%), and 0.500 mm (30%) stents. The reduction in longitudinal retraction with increasing strut diameter indicates that thicker struts provide greater resistance to axial deformation during stent deployment. This behavior is primarily attributed to the increase in axial and bending stiffness, which limits the rotation and deformation of the rhombic cells under radial expansion. As a result, the stent undergoes smaller longitudinal dimensional changes during deployment. From a clinical perspective, lower longitudinal retraction is desirable because excessive axial deformation may compromise deployment accuracy and increase the likelihood of localized mechanical interaction with the arterial wall. Therefore, increasing the strut diameter improves the longitudinal stability of the stent. However, this improvement should be balanced against the increase in dog-boning observed for thicker-strut designs, highlighting the inherent trade-off in stent optimization.

These results demonstrate a design trade-off in rhombic-cell stents. Increasing the strut diameter reduces elastic recoil, foreshortening, and longitudinal retraction, thereby enhancing the dimensional stability of the stent after deployment. However, these improvements are accompanied by a noticeable increase in the dog-boning effect. Therefore, selecting an appropriate strut diameter requires balancing deployment uniformity against post-deployment mechanical stability. To systematically assess the influence of strut diameter on stent performance, five mechanical performance indicators were considered: diametral strain, elastic recoil, dog-boning, foreshortening, and longitudinal retraction. These indicators characterize the key aspects of stent deployment, including expansion efficiency, recoil behavior, deployment uniformity, and dimensional stability after deployment. Qualitative comparison of these metrics identifies the trade-off among competing mechanical characteristics and supports comparison of the investigated strut diameters.

The adopted assumptions also affect the absolute deployment metrics. Without the arterial wall and blood pressure, radial constraint during recoil differs from vessel supported deployment; the ideal cylindrical balloon can alter end expansion and dog-boning; and the elastoplastic PLA/PDO representation does not include viscoelastic rate dependence over the 1 s loading cycle. The absolute values should therefore be interpreted within these numerical assumptions, while the comparison among strut diameters reflects the controlled geometric trends of the present model.

Table 4 provides a qualitative synthesis of the five performance indicators based on the relative trends observed among the four configurations. No formal weighting or multicriteria decision method was applied; therefore, the table is intended only to show the geometric trade-off. Within this qualitative comparison, the 0.35 mm strut diameter shows a balanced response, combining good expansion capability, low recoil, moderate dog-boning, and high longitudinal stability. The star ratings in Table 4 represent only relative qualitative performance among the four investigated configurations and should not be interpreted as weighted numerical scores.

## 5. Conclusions

The present study demonstrates that the strut diameter is a key geometric parameter influencing the mechanical performance of rhombic-cell biodegradable stents. Increasing the strut diameter progressively enhances post-deployment dimensional stability by reducing elastic recoil, foreshortening, and longitudinal retraction, while simultaneously improving radial stability. However, these benefits are accompanied by a progressive increase in the dog-boning effect, indicating less uniform radial expansion during deployment. These findings highlight an inherent design trade-off between post-deployment mechanical stability and deployment uniformity. Therefore, the selection of a suitable strut diameter should be based on achieving a balanced compromise among these competing mechanical characteristics rather than optimizing a single performance metric.

Among the investigated designs, the stent with a 0.35 mm strut diameter exhibited the most balanced response within the qualitative comparison used in this study. Compared with thinner struts, it substantially reduced elastic recoil, foreshortening, and longitudinal retraction, while avoiding the pronounced dog-boning observed in the 0.50 mm design. Consequently, within the four configurations investigated and under the adopted numerical assumptions, the 0.35 mm configuration represents a balanced geometric trade-off rather than a formally optimized design. These conclusions should therefore be interpreted as comparative design trends within the present numerical framework rather than as direct predictions of clinical performance.

## Figures and Tables

**Table 1:** Material properties used in the finite element model.

	Density (kg/m^3^)	Young’s Modulus (MPa)	Poisson Ratio	Material Model	Ref.
Stent PLA/PDO (60/40)	1470	1210	0.37	Elastoplastic	[13]
Balloon	1100	920	0.40	Linear elastic	[34]

**Table 2:** Dimensions of the stent.

	Outer Diameter Do (mm)	Length LS (mm)	Inner Diameter Di (mm)	Thickness t (mm)	Number of Peaks np	Number of Revolutions nf
Stent 1	3	20	2.7	0.150	4	1
Stent 2	-	-	2.5	0.250	-	-
Stent 3	-	-	2.3	0.350	-	-
Stent 4	-	-	2	0.500	-	-

**Table 3:** Dimensions of the balloon.

	Deflated Condition		Inflated Condition		Recoiled Condition	
	Diameter (mm)	Length (mm)	Diameter (mm)	Length (mm)	Diameter (mm)	Length (mm)
Balloon 1	2.5	25	5.5	25	2.5	25
Balloon 2	2.3	-	5.3	-	2.3	-
Balloon 3	2.1	-	5.1	-	2.1	-
Balloon 4	1.9	-	4.9	-	1.9	-

**Table 4:** Qualitative Synthesis of Mechanical Performance for the Investigated Strut Diameters.

Strut Diameter	Expansion Capability	Radial Stability (Low Recoil)	Deployment Uniformity (Low Dog-Boning)	Longitudinal Stability (Low Foreshortening)	Axial Stability (Low Longitudinal Retraction)	Overall Performance
0.15 mm	★★★★☆	★☆☆☆☆	★★★★★	★☆☆☆☆	★☆☆☆☆	Poor
0.25 mm	★★★★☆	★★★☆☆	★★★★☆	★★★☆☆	★★★☆☆	Good
0.35 mm	★★★★☆	★★★★☆	★★★☆☆	★★★★☆	★★★★☆	Balanced
0.50 mm	★★★★★	★★★★★	★☆☆☆☆	★★★★★	★★★★★	Very Good, but High Dog-boning

Note: ★ and ☆ represent the relative qualitative performance on a five-star scale; a greater number of ★ indicates better relative performance among the investigated configurations.

## Data Availability

The data that support the findings of this study are available on request from the authors.
